# Bioorthogonal Caging-Group-Free Photoactivatable Probes for Minimal-Linkage-Error Nanoscopy

**DOI:** 10.1021/acscentsci.3c00746

**Published:** 2023-07-26

**Authors:** Ayse Aktalay, Richard Lincoln, Lukas Heynck, Maria Augusta do R. B. F. Lima, Alexey N. Butkevich, Mariano L. Bossi, Stefan W. Hell

**Affiliations:** † Department of Optical Nanoscopy, Max Planck Institute for Medical Research, Jahnstraße 29, 69120 Heidelberg, Germany; ‡ Department of NanoBiophotonics, Max Planck Institute for Multidisciplinary Sciences, Am Fassberg 11, 37077 Göttingen, Germany

## Abstract

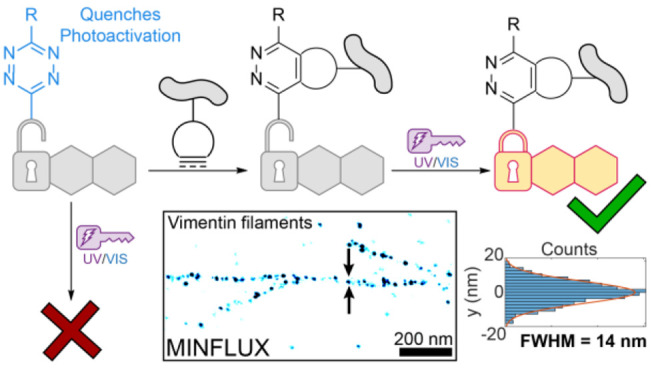

Here we describe highly compact, click compatible, and photoactivatable dyes for super-resolution fluorescence microscopy (nanoscopy). By combining the photoactivatable xanthone (PaX) core with a tetrazine group, we achieve minimally sized and highly sensitive molecular dyads for the selective labeling of unnatural amino acids introduced by genetic code expansion. We exploit the excited state quenching properties of the tetrazine group to attenuate the photoactivation rates of the PaX, and further reduce the overall fluorescence emission of the photogenerated fluorophore, providing two mechanisms of selectivity to reduce the off-target signal. Coupled with MINFLUX nanoscopy, we employ our dyads in the minimal-linkage-error imaging of vimentin filaments, demonstrating molecular-scale precision in fluorophore positioning.

## Abstract 2

The quenching properties of tetrazine are used to control the photoswitching of fluorophores, yielding highly compact, click compatible, photoactivatable dyes for super-resolution microscopy.

Advancements in live-cell super-resolution fluorescence microscopy (nanoscopy) methods beget greater demands on the fluorophores and labels used for imaging, to fully leverage the improvements in optical resolution.^1,2^ To distinguish adjacent fluorophores at molecular-scale proximities, super-resolution methods rely on the sequential “on”–“off” transitioning of fluorophores to generate distinct molecular states. This is commonly achieved with cyanines^3^ in complex imaging buffers that are incompatible with live-cell imaging.^4^ Cyanines further suffer from intermolecular energy transfer between dark states, limiting the molecular distances that can be separated.^5^ These constraints may be addressed by employing alternative stochastic blinking fluorophores,^6−12^ photoswitchable dyes,^13,14^ or photoactivatable (caged) dyes.^15−17^

Furthermore, as the improvements in obtainable optical resolution reach single nanometer precision, as it is the case for MINFLUX (minimal photon fluxes)^18−21^ and MINSTED nanoscopy techniques,^22,23^ the displacement of the fluorophore from the point of interest (referred to as linkage error) becomes a critical parameter.^1,24^ Genetic code expansion (GCE) has emerged as a powerful platform for site-specific labeling of proteins with small organic fluorophores with minimal linkage error.^2,25^ Through incorporation of unnatural amino acids (UAAs) with bioorthogonal reactivity to fluorescent probes, proteins of interest can be labeled in a fast and specific manner.^26^ The most prominent example is the combination of “clickable” UAAs and tetrazine-functionalized fluorophores for highly specific labeling by live-cell compatible strain-promoted inverse electron-demand Diels–Alder cycloaddition (SPIEDAC) reactions (Figure 1).^27^ A further advantage of tetrazine-fluorophore dyads is fluorogenicity, which arises if the fluorescence signal is partially or fully quenched by the unreacted 1,2,4,5-tetrazine (Tz) fragment^28^ and then restored upon reaction with the strained alkyne.^29^ This “turn-on” effect has been extensively utilized to develop labels for no-wash imaging.^10,30−32^ Despite their significant potential, only a handful of Tz-fluorophore dyads are compatible with fluorescence nanoscopy methods with sufficient optical resolution to observe the advantages in reduced linkage error,^2,9,25^ and fewer still are commercially available.^5^ To date, no Tz-fluorophore dyads have been utilized to label UAAs for MINFLUX imaging—the closest example is an azide plus variant of a cyanine dye (similar to Alexa Fluor 647), in combination with copper-mediated click chemistry, and a dedicated buffer to achieve blinking.^25^

**Figure 1:**
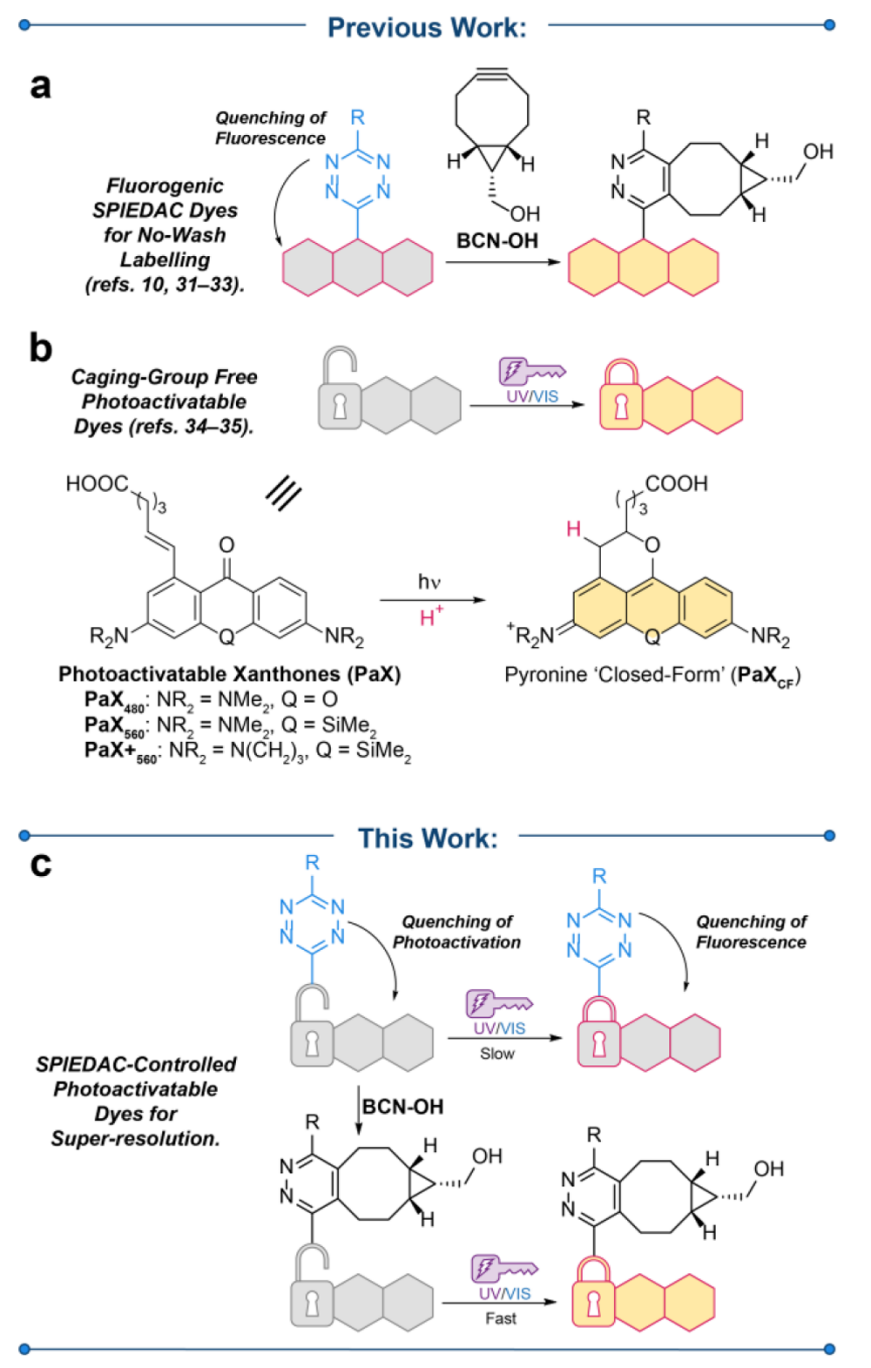
(a–c) Rational design of a photoactivatable tetrazine dyad for super-resolution microscopy. Previous efforts to build fluorogenic labeling strategies utilizing (a) strain-promoted inverse electron demand Diels–Alder (SPIEDAC) reactions and (b) caging-group free photoactivatable fluorophores, including photoactivatable (PaX) dyes which undergo photoactivation to a highly fluorescent “closed form” (PaX_CF_). This work (c) utilizes SPIEDAC to control both the brightness and the switching rates of photoactivatable fluorophores for nanoscopy.

We recently reported a new class of caging-group-free photoactivatable xanthone (PaX) analogues that upon UV (one photon) or NIR (two photon) irradiation convert rapidly and cleanly into highly fluorescent pyronine dyes in a triplet mediated reaction.^33,34^ Due to their small, uncharged structures, these PaX labels can be used for fixed or live-cell STED (stimulated emission depletion) and PALM (photoactivated light microscopy) as well as MINFLUX nanoscopy. We reasoned that tetrazine moieties, in addition to imparting fluorogenicity to UAA-specific labels, will further attenuate the rate of photoactivation of the PaX core by quenching of the xanthone excited state—precluding intersystem crossing and subsequent fluorophore formation. Thus, tetrazine functionalization of PaX dyes constitutes a suitable platform to construct compact, high-contrast photoactivatable labels with minimal linkage errors that are ideally suited for MINFLUX nanoscopy.

We report herein a series of PaX dyads incorporating tetrazine moieties (PaX-Tz) for nanoscopy imaging, in which we systematically varied the xanthone analogue, tetrazine, and linking strategy between the two in order to modulate photoactivation rates and the fluorescence contrast between the unreacted tetrazine (Tz) and reacted pyridazine (Pz) adducts of the pyronine fluorophore (Figure 1). The quenching imparted by the tetrazine is eliminated upon reaction with the target UAA, resulting in faster photoactivation and brighter fluorescence following labeling. Finally, we demonstrated the utility of these labels for STED, PALM, and MINFLUX nanoscopy of vimentin filaments incorporating UAAs for truly molecular-scale visualization of protein–protein distances.

## Results and Discussion

### Synthesis and Characterization of PaX Tetrazine Labels

A number of diversely functionalized tetrazines have been reported, with improved rates of reactivity to UAAs, typically anticorrelated with their chemical stabilities.^27,35,36^ We reasoned that an ideal combination of tetrazine (Tz) and photoactivatable xanthone (PaX) would result in quenching of the singlet excited state of the xanthone, via an energy transfer mechanism analogous to quenching observed in fluorescent tetrazine dyads,^37^ precluding intersystem crossing and, in turn, photoassembly of the fluorophore.

**Figure 2:**
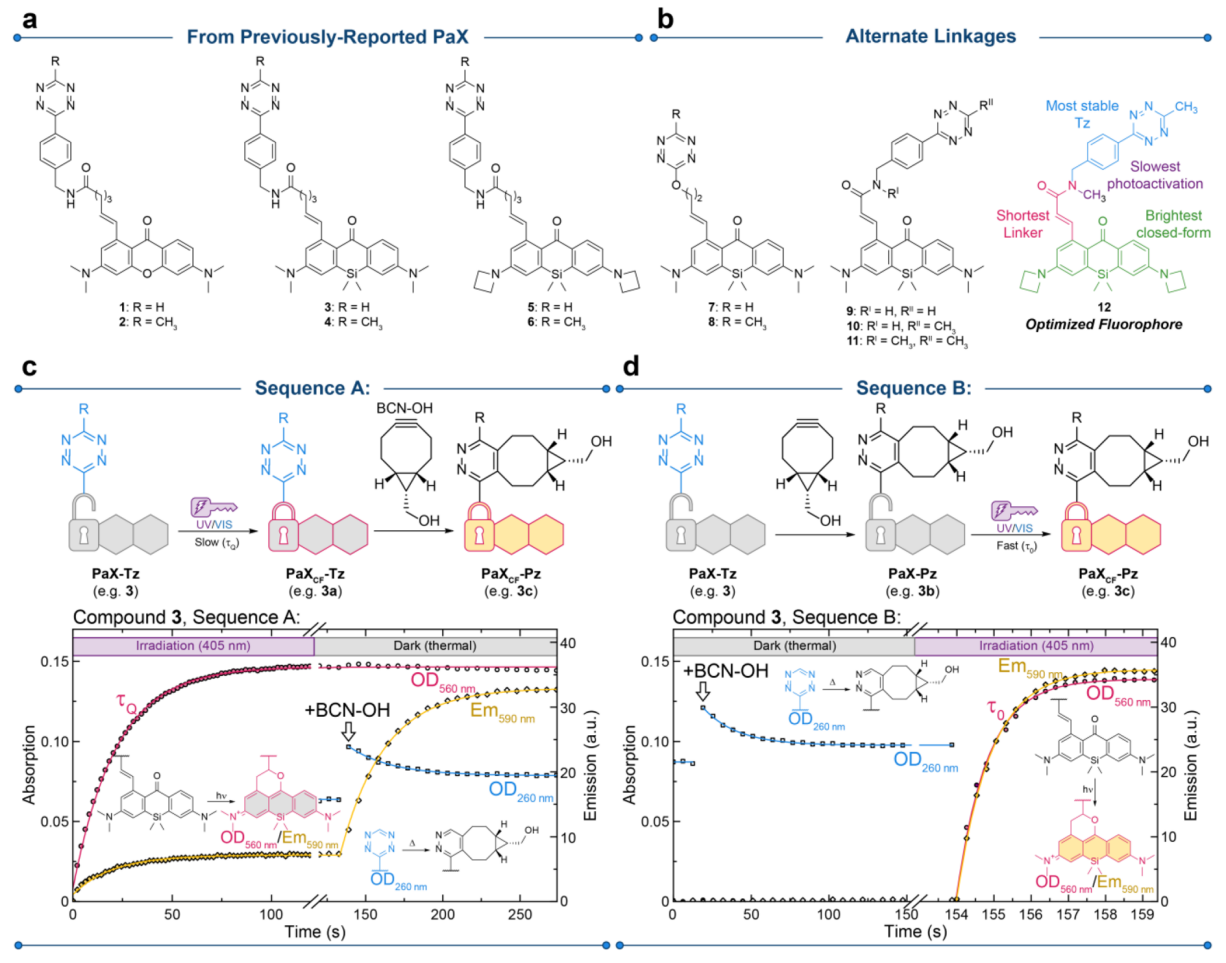
Structures and characterization of PaX-Tz dyads. (a) Chemical structures of the PaX-Tz dyads studied in this work derived from previously reported PaX.^33^ (b) Alternate linkage designs. (c) Effect of the Tz moiety on the photoactivation behavior of PaX-Tz dyes, exemplified with compound **3**. In Sequence A, the temporal evolution of the absorption and fluorescence spectra of **3** (**3** → **3a**; 1.66 μg mL^–1^) irradiated in methanol (λ_act_ = 405 nm) was monitored. BCN–OH (50 equiv) was subsequently added, and the effect of Tz-quenching of the fluorescence was observed (**3a** → **3c**). (d) In Sequence B, BCN–OH was first allowed to react with **3** (**3** → **3b**), then following the temporal evolution of the absorption and fluorescence spectra upon irradiation (**3b** → **3c**).

In order to identify such a combination of PaX and Tz moieties, we synthesized a series of PaX-Tz dyads (Figure 2a). Constructed from the reported PaX carboxylic acids (PaX_480_, PaX_560_, and PaX+_560_),^33^ we first varied the diarylketone between an electron-rich oxygen-bridged amino-xanthone (PaX_480_; **1**, **2**) and electron-deficient silicon-bridged xanthones (PaX_560_, PaX+_560_) bearing either dimethylamine (**3**, **4**) or azetidine auxochromic groups (**5**, **6**) in order to elucidate electronic effects on quenching. We next varied the Tz moiety, selecting either the more-reactive unsubstituted (**1**, **3**, and **5**) or more shelf-stable methyl-substituted (**2**, **4**, and **6**) phenyl tetrazines. We further explored alternative linkage strategies between the Tz unit and the PaX (Figure 2b), in order to reduce the linkage error and the influence of distance on quenching.^37^ The first strategy involved ether-linked dyads (**7**, **8**), derived from the corresponding 3-bromo-1,2,4,5-tetrazine^38^ and PaX_560_ analogue with an alcohol-terminated linker. However, we found that such constructs suffered from poor reactivity of the Tz moiety and were susceptible to hydrolytic cleavage of the Tz unit. In our second strategy, we constructed a PaX (**S9**, see Supporting Information) with an acrylate moiety as the linker/radical trap, which served to generate a series of the dyads bearing either secondary (**9**, **10**) or tertiary (**11**, **12**) acrylamide linkages. Initial TD-DFT studies^28^ anticipated excited state energy transfer to the Tz moiety in both the singlet and triplet manifolds for the PaX structures, regardless of linkage (Figure S1a), as well as quenching of the singlet excited state of the photogenerated (i.e., “closed-form”) pyronine fluorophore (PaX_CF_, Figure 1b and Figure S1b). Recording the fluorescence lifetime of compound **4** at 490 nm before photoactivation showed significant shortening of the lifetime compared to PaX_560_ (Figure S2a), further affirming the ability of the Tz moiety to quench the singlet state of the diaryl ketone and, in turn, prevent photoswitching from the triplet state. After the photoactivation of **4**, the fluorescence lifetime of the corresponding pyronine (**4a**) at 585 nm was also significantly shorter than that of the fluorophore photogenerated from PaX_560_, emphasizing a potential fluorogenic response (Figure S2b).

To evaluate the effect of the Tz moiety on the photoactivation of the PaX chromophore, irradiation experiments were first conducted in methanol, prior to and after complete reaction of the Tz moiety with (1*R*,8*S*,9*s*)-bicyclo[6.1.0]non-4-yn-9-ylmethanol (BCN–OH) to give the corresponding pyridazine (Pz) product. In the first experimental sequence (Sequence A, Figure 2c) the dyads were irradiated with 405 nm light until complete conversion to the corresponding “closed-form” (PaX_CF_; e.g., **3a**, see Figure S3a), which was monitored spectroscopically at the absorption and emission maxima of the PaX_CF_ (560 and 590 nm, respectively, for compound **3**). This established the rate of photoactivation in the presence of Tz quenching (i.e., the rate of conversion from PaX-Tz to PaX_CF_-Tz). Excess BCN–OH (50 equiv) was next added, resulting in an enhancement of the fluorescence signal (590 nm for **3a** to **3c**) concomitant with a decrease in absorption from the Tz (260 nm) as the SPIEDAC reaction occurred. In the second experimental sequence (Sequence B, Figure 2d), the dyads were first allowed to react completely with BCN–OH yielding the pyridazine dyad (PaX-Pz; e.g., **3b**, see Figure S3a), and subsequently photoactivated to the “closed-form” (PaX_CF_-Pz), establishing the rate of photoactivation in the absence of the Tz moiety. Figure S3b shows the spectral changes corresponding to Sequences A and B for compound **3**, and Figure S4 shows the LC-MS analysis for intermediates **3a**–**3c**.

**Figure 3:**
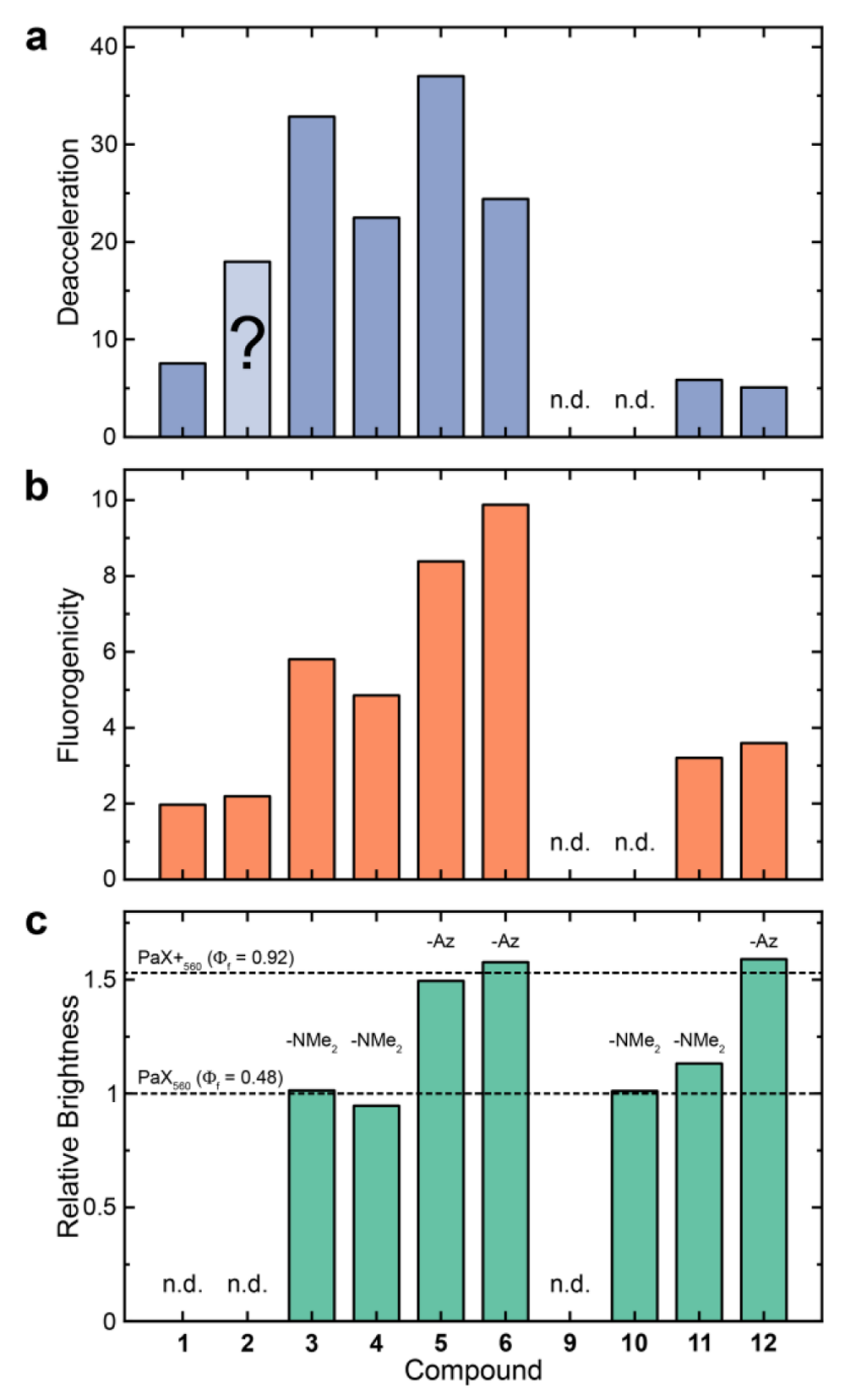
(a) Effect of Tz moiety on the photoactivation rate (i.e., deacceleration) in the Tz dyads calculated from that ratio of photoactivation rates from Sequence B vs Sequence A for each dyad measured in methanol. The ratio could not be determined for compounds **9** and **10** and is ambiguous for compound **2** due to photobleaching during Sequence A (denoted with a question mark). (b) Effect of Tz moiety on the ratio of fluorescence emission quantum yields (i.e., fluorogenicity) of the closed-form products PaX_CF_-Pz and PaX_CF_-Tz from Sequence B and Sequence A, respectively, for each dyad measured in methanol (could not be determined for compounds **9** and **10**). (c) Relative brightness for the PaX_CF_-Pz (from sequence B) for each dyad compared to the free acids PaX_560_ and PaX+_560_.

The designed experiment allows for a quantitative comparison of the relevant properties for the presented dyads. The ratio of the photoactivation speed of the PaX-Tz and PaX-Pz (deacceleration) provides a measure of the Tz quenching of the photoactivation process for all the dyads (Figure 3a). Additionally, the ratio of apparent fluorescence quantum yields of PaX_CF_-Tz and PaX_CF_-Pz provided a measure of the fluorogenicity of the compounds (Figure 3b) and allowed a comparison of quantum yields across the family of PaX_CF_ (Figure 3c). Lastly, LC-MS analysis was performed on the reaction mixtures and revealed no major byproducts or photoproducts of Tz quenching unless otherwise indicated (Figure S5).

In general, we observed an increase in photoactivation speed after reaction with BCN–OH, confirming quenching of the photoactivation pathway by the Tz moiety, and an approximately 5-fold fluorogenic response of the PaX_CF_-Tz upon reaction with BCN–OH. More specifically, for the compounds derived from PaX_480_ (**1**, **2**, see Figure S6) the combination of slower photoactivation rates and lower photostability of the dye scaffold, when combined with the quenching of Tz, resulted in significant oxidative photoblueing^39,40^ from the prolonged irradiation over the course of Sequence A (Figure S6a–d), which was further confirmed in the LC-MS analysis (Figure S6e,f). This was most notable for compound **2** where the demethylated product was primarily observed. Compound **4** and the dyads derived from the azetidine functionalized PaX+_560_ compounds (**5**, **6**) yielded comparable results to compound **3**, with compound **5** showing the greatest quenching of photoactivation and **6** showing the greatest fluorogenicity of all of the dyads.

Changing the linkage strategy to an ether (**7**, **8**) enabled shortening the Tz–PaX distance; however, insufficient solvolytic stability of the ether resulted in slow loss of the Tz in methanol (Figure S7a), precluding their study by Sequence A. Both compounds **7** and **8** reacted with BCN–OH, and the resulting product was resistant to methanolysis (Figure S7b). Photoactivation of the dyad after reaction with BCN–OH was rapid and clean (Figure S7c,d); however, the electrophilicity of the alkoxytetrazines may preclude their use in live-cell experiments.

To prepare the acrylamide-linked PaX scaffolds (**9**–**12**), new acrylate-functionalized PaX were prepared (**S9**, see Supporting Information for details) and coupled to commercially available tetrazine amines to yield the secondary acrylamide products (**9**, **10**). To access the tertiary *N*-methylacrylamides (**11**, **12**), *N*-methyl-4-(6-methyl-1,2,4,5-tetrazin-3-yl)benzylamine^41^ was additionally synthesized and coupled with the acrylic acids **S9** and **S14** (see Supporting Information). Much to our surprise, the acrylamide-derived PaX scaffolds showed notably slower photoactivation rates, likely due to the greater electron deficiency of the radical trap. After photoactivation, the secondary acrylamides established an equilibrium with the products of nucleophilic addition of a methanol solvent (Figure S8 for Compound **9**). For compound **10**, this was particularly apparent, with the primary photoproduct of Sequence A being nonabsorbing at 570 nm and nonfluorescent. In Sequence B (following the reaction with BCN–OH), the photoactivation was fast and yielded the desired **10c**, making **10** a promising candidate for no-wash labeling. Due to these complications, the effect of Tz on the photoactivation rate could not be reliably determined for compounds **9** and **10**, but tertiary acrylamide-functionalized PaX scaffolds represent a useful general strategy to reduce the photoactivation rate of PaX fluorophores.

With the reduced linker length of the acrylamides **9**–**12**, we anticipated better quenching between the Tz and PaX. However, we observed only modest fluorogenicity and photoactivation suppression for the tertiary acrylamides **11** and **12**. This may be due to the reduced flexibility of the acrylamide linker, hindering the adoption of a π-stacked geometry that brings the Tz close to the pyronine fluorophore, as compared to the dyads with longer linkers. Importantly, the acrylamide had no effect on the fluorescence quantum yield (Φ_f_) of the PaX_CF_-Pz, with the Φ_f_ ultimately being determined by the nature of *N*,*N*-dialkylamino substituents (dimethylamino vs. 1-azetidinyl (-Az) auxochrome; see Figure 3c). Compound **12** provided the ideal combination of properties for nanoscopy: an azetidine-bearing PaX scaffold for the highest Φ_f_ of the "closed-form" (**12c**); a secondary acrylamide linker for minimal size and slowest photoactivation, both before and after reaction with BCN–OH (for greater activation control during imaging); and a methyl-functionalized tetrazine for improved shelf life.

To determine if the fluorogenic effect and reactivity of our dyads translated into a more biologically relevant setting, the methyl-functionalized dyads (**4**, **6**, **8**, and **10**–**12)** were further studied in buffered solutions containing FBS (fetal bovine serum) at the concentration used in our cell culture medium (10% v/v), with *endo* BCN-*L*-lysine as the model alkyne (BCN–OH was not soluble in this medium at the required concentrations). Unlike in the experiments performed in methanol, the high protein concentration (3.9 g/100 mL) precluded monitoring the reaction with *endo* BCN-*L*-lysine (due to a spectral cutoff at 290 nm) and LC-MS data analysis. Therefore, the dyads were allowed to react for a long period of time with *endo* BCN-*L*-lysine (over 50–100 min) to ensure complete reaction. The photoconversion to the “closed-form” (PaX_CF_-Pz) was then followed based on the absorption at 560 nm. These experiments (Figure S9) demonstrate the ability of all studied compounds to undergo conjugation and photoactivation in aqueous environments and allow the observation of the effect of the Tz moieties on the activation rates (10–20 fold deacceleration) and fluorogenic characteristics (3–9 fold fluorogenicity) of the labels (Figure S10), with similar tendencies as observed in methanol. While acrylamide derivatives (**10**–**12**) display lower deacceleration and fluorogenicities than alkyl-chain derivatives, the photoactivation rates of their pyridazine products are around 4-fold slower compared to the alkyl-substituted analogues.

In summary, the following conclusions can be drawn: (i) the electron-deficient Si-PaX (**3**, **4**) had greater deacceleration and higher fluorogenicity than the electron-rich O-PaX (**1**, **2**); (ii) the more chemically reactive H-Tz’s (**3**, **5**) provided better quenching of the photoactivation process and were slightly less fluorogenic than their methylated analogues (**4**, **6**); (iii) the dyads with ether-linked Tz’s (**7**, **8**) and secondary acrylamides (**9**, **10**) demonstrated higher electrophilic reactivity; (iv) the alkenyl PaX’s (**3**–**6**) experienced greater deacceleration and fluorogenicity than the tertiary acrylamide PaX’s (**11**–**12**) despite their much faster photoactivation rate.

**Figure 4:**
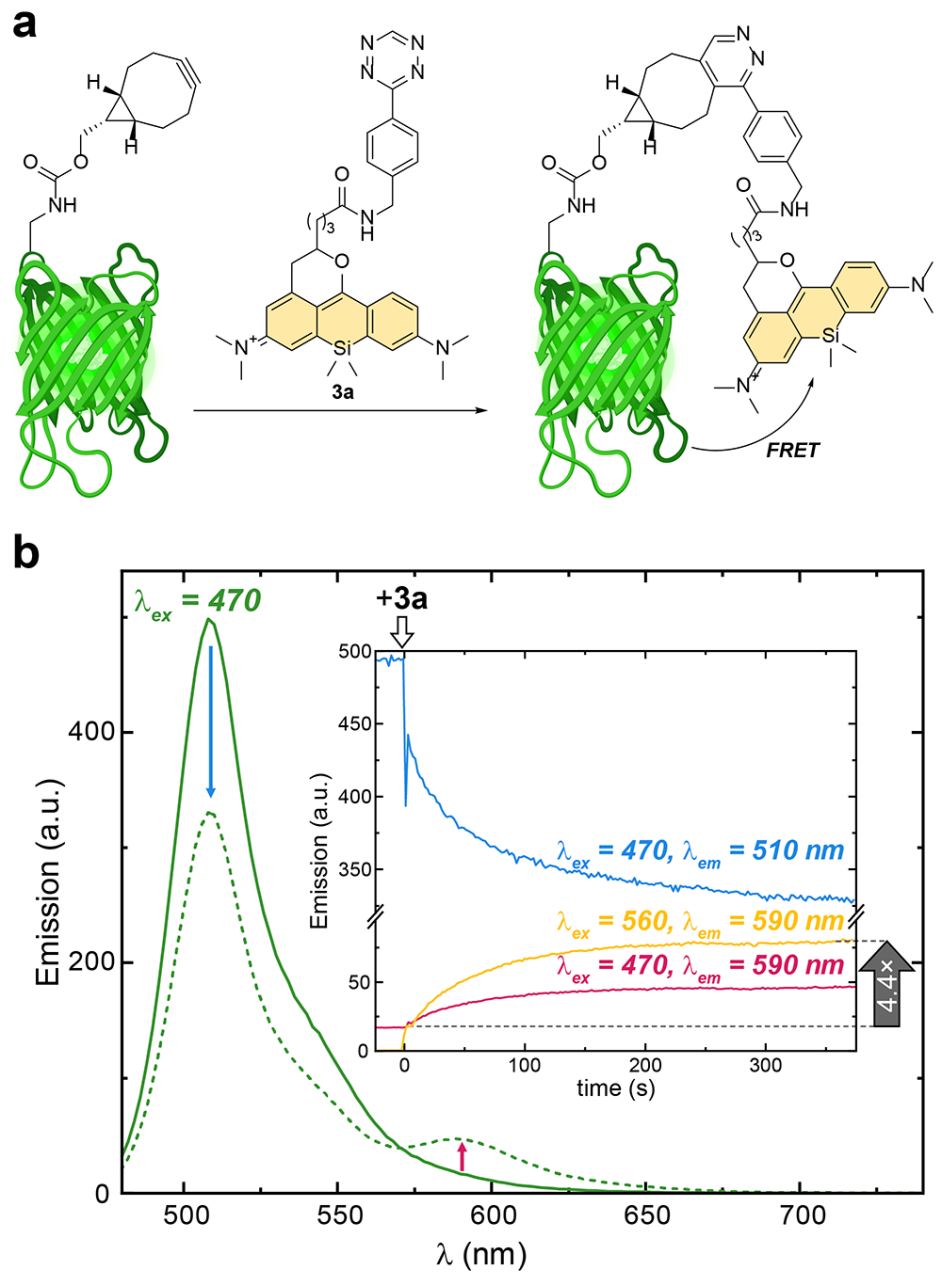
Demonstration of FRET in GFP labeled with a PaX-Tz fluorophore. (a) Schematic representation of GFP-PaX dyad. GFP protein (1.1 eq.; for plasmid details, see the Supporting Information) containing *endo* BCN-*L*-lysine^42−46^ was reacted with **3a** in PBS at pH 7.4. (b) Fluorescence spectra of GFP before (solid line) and after (dashed line) reaction with **3a**. Inset: Temporal evolution of the fluorescence signal upon addition of **3a**, indicative of FRET from GFP to compound **3a** upon SPIEDAC reaction.

Finally, as a first demonstration of the close proximity labeling of a target protein, we reacted compound **3a** (prepared by photolysis of compound **3**) with a green fluorescent protein (GFP) variant bearing a Y35TAG point mutation for the incorporation of *endo* BCN-*L*-lysine (Figure 4a).^42−46^ By monitoring the fluorescence of the GFP upon excitation at 470 nm, as well as the new emission band at 590 nm, we could observe Förster resonance energy transfer (FRET) from the GFP (donor) to the PaX_CF_ (acceptor; see Figure 4b). Furthermore, by monitoring the emission of PaX_CF_ at 590 nm while directly exciting with 560 nm light, we could quantify the fluorescence enhancement of the fluorophore as the tetrazine moiety reacted with BCN, observing a 4.4-fold enhancement in fluorescence, consistent with previous experiments. Mass spectrometry confirmed covalent labeling with **3a** (Figure S11).

### Bioorthogonal Probes for Fluorescence Nanoscopy

**Figure 5:**
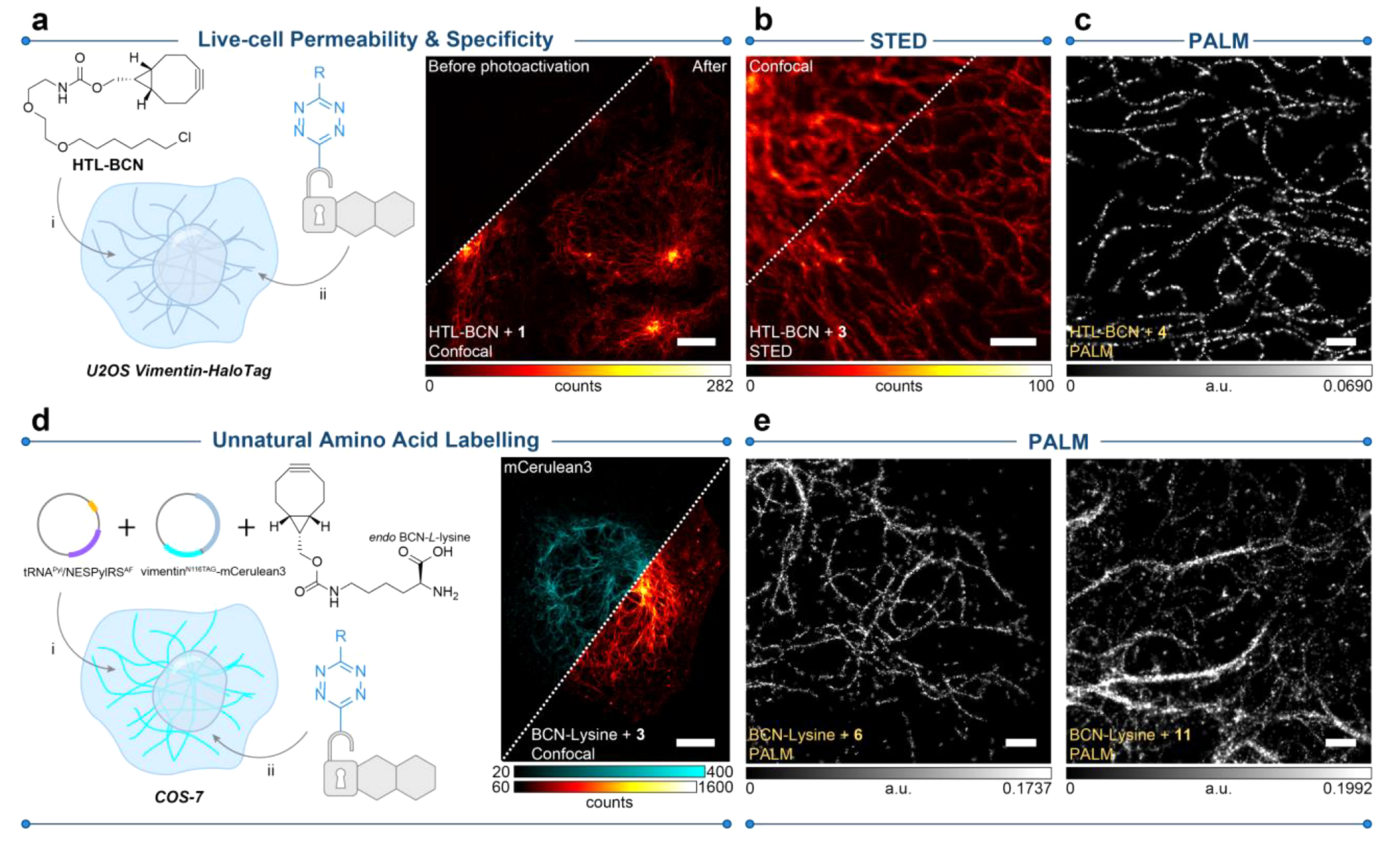
Live-cell labeling and imaging with PaX-Tz dyads. (a) Live cell permeability and BCN labeling specificity were assessed by confocal imaging in U2OS cells stably expressing a vimentin-HaloTag construct in a two-step labeling strategy utilizing the HaloTag specific ligand HTL-BCN (10 μM, 30 min) and a dyad (200 nM, overnight), fixed with PFA and mounted in PBS (e.g., **1**, right panel). Conversion to PaX_CF_ was achieved by a 405 nm illumination. (b) STED image of cells described in **a**, labeled with compound **3**. Conversion to PaX_CF_ was achieved with widefield illumination (AHF analyzentechnik AG, DAPI filter set F46–816). (c) PALM image of cells described in panel a, labeled with compound **4**. (d) COS-7 cells transiently expressing a vimentin-mCerulean3 fusion construct^49^ carrying a N116TAG mutation (for incorporating the UAA *endo* BCN-*L*-lysine) in vimentin.^50,51^ The plasmids were provided by the Lemke laboratory (EMBL, Heidelberg). See Figure S14 for detailed maps of tRNAPyl/NESPylRSAF, and vimentin^N116TAG^-mCerulean3 plasmids. Transfected cells were labeled with a dyad (500 nM, 4 h), fixed with PFA and imaged. Right: confocal image of a transfected cell showing mCerulean signal (cyan) and colabeled with **3** (red hot). Conversion to the PaX_CF_ was achieved by 405 nm illumination. (e) PALM imaging of cells described in panel d labeled with compounds **6** (left) and **11** (right). Scale bars: 10 μm (a, d), 500 nm (c, e).

We next turned our attention to the application of PaX-Tz dyads in fluorescence microscopy and nanoscopy studies. In order to test the permeability and specificity in live-cell labeling, we initiated our studies utilizing a two-step labeling of HaloTag-expressing cells.^10,47^ In this protocol, U2OS cells stably expressing a vimentin-HaloTag construct^48^ were first labeled with the chloroalkane ligand HTL-BCN (Figure 5a), following an established protocol (10 μM, 30 min) to incorporate the cycloalkyne into the HaloTag, followed by the overnight labeling with the respective dyad (**1**–**12**, 200 nM). Afterward, the cells were fixed with paraformaldehyde and imaged by confocal microscopy (Figure 5a and Figure S12). For most dyads, the labeling was specific for vimentin filaments, and showed a high contrast between images recorded before and after photoactivation. Alkoxytetrazine (**7**, **8**) and secondary acrylamide (**9**, **10**) dyads failed to give meaningful images, which may be attributed, for the former, to the slower reaction kinetics and hydrolysis of alkoxytetrazines and, for the latter, to the side reactivity of secondary acrylamides with cellular nucleophiles. However, the additional methyl group in the tertiary acrylamides sufficiently blocked this unwanted reactivity in compounds **11** and **12**.

In our previous work, PaX-derived probes (such as PaX_560_ conjugated antibodies and nanobodies) were compatible with STED nanoscopy, which we confirmed was also the case for clickable Tz dyad **3** using depletion at 660 nm, giving results consistent with our previous report (Figure 5b). Acrylamide-derived dyads were, however, incompatible with STED, which may be due to their red-shifted absorption closer to the depletion wavelength and lower fluorophore fatigue resistance. All dyads with good labeling (i.e., excluding **7**–**10**) performed remarkably well in PALM (Figure 5c and Figure S13), where we observed well-resolved structures. As expected from their photophysical properties, compounds **5**, **6**, and **12** bearing azetidine auxochromic groups yielded higher overall photon counts. Methyl substituted Tz derivatives (**2**, **4**, **6**) yielded 15–20% less photons than the unsubstituted analogues (**1**, **3**, **5**). We also observed that tertiary acrylamide-PaX (**11**, **12**) required a higher intensity 405 nm photoactivation in PALM, while also yielding more photons than their first-generation counterparts (**4**, **6**). Taken together, these results highlight the diverse applicability of PaX-Tz dyads: while the probes featuring alkene radical traps (e.g., **3**) require much less UV activation, they are STED-compatible; tertiary acrylamides **11** and **12** afford much greater control in localization-based techniques.

Next, COS-7 cells were prepared (Figure 5d), transiently expressing a vimentin-mCerulean3 fusion construct^49^ carrying a N116TAG mutation (for incorporating the UAA *endo* BCN-*L*-lysine, Figure S14) in the 1A coil fragment of the vimentin head domain.^50,51^ Initial staining with **3** (Figure 5d) demonstrated bright labeling of the mCerulean3-tagged vimentin filaments after photoactivation, with a minimal background that can be attributed to nonincorporated UAA, free or bound to tRNA, or unreacted PaX-Tz in lipophilic compartments. Using this strategy, cells were further imaged by PALM microscopy (Figure 5e and Figure S15) for a subset of our dyads.

Based on the imaging results and the above spectroscopic characterizations, we anticipated that compounds **3**–**6** and **11**–**12** will be compatible for MINFLUX with 560 nm excitation. We selected compounds **11** and **12** for subsequent MINFLUX experiments to yield a higher control of the photoactivation process during imaging.

### Minimal Linkage Error MINFLUX Nanoscopy of Vimentin Filaments

**Figure 6:**
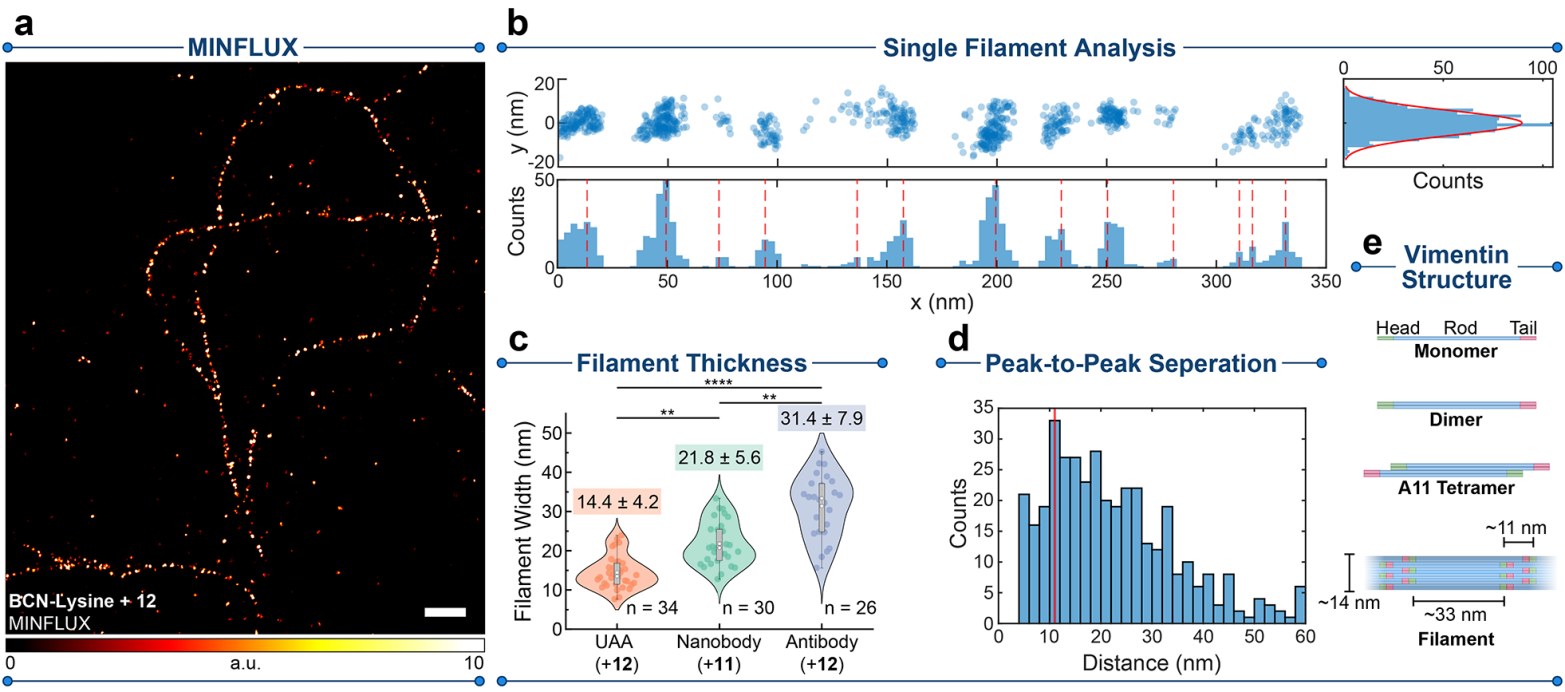
MINFLUX characterization of linkage-error-free labeling. (a) MINFLUX image of vimentin filaments in COS-7 cells incorporating *endo* BCN-*L*-lysine as described in Figure 3d and labeled with compound **12**. (b) Single filament analysis of the linearized filament marked in panel a; distributions of localizations along the filament length (x) and width (y) were used to determine peak-to-peak separation of localization domains and the fwhm of the filament segment, respectively. (c) Distribution of filament thicknesses of vimentin segments labeled with different strategies, including UAAs + compound **12**, anti-GFP nanobodies + compound **11**, and indirect immunofluorescence + compound **12** in transfected COS-7 cells (as described above). The distribution mean ± SDs are given above the violin plots, and n designates the number of analyzed filaments segments. **P < 0.01; ****P < 0.0001 using the Kruskal–Wallis and the Dunn method for posthoc correction for multiple comparisons. (d) Peak-to-peak separations of localization domains in vimentin filaments labeled with UAAs. (e) Structure of the vimentin according to recent literature with our obtained values shown.^56^ Scale bar: 200 nm.

To demonstrate the advantage of linkage-error-free labeling with our dyads, we turned to MINFLUX nanoscopy to visualize the vimentin filaments prepared via GCE (as described above) and stained with compound **12**. Individual fluorophores were localized by MINFLUX with a median precision of 2–3 nm, utilizing 100 photons per localization (Figure 6a and Figure S16). Measuring the average full-width half-maximum (fwhm) of linearized filament segments (Figure 6b, see Figure S17 and accompanied Supporting Information for methodology), we obtained an average value of 14 nm, which was in excellent agreement with the value reported by cryogenic electron microscopy/electron tomography data,^52−55^ thus confirming that the proposed PaX-Tz labeling strategy reduces the linkage error, in practice, to an undetectable level.

To support this claim, we also recorded as a comparison MINFLUX images of the transfected cells labeled with nanobodies targeting the mCerulean3 and carrying the Tz dyad **11** attached via TCO-PEG3-maleimide coupling (Figure S18). Based on MS analysis, using compound **12** resulted in lower quality labeling (Figure S18), and the resulting nanobodies were prone to precipitation over long-term storage, likely due to lower aqueous solubility imparted by the azetidine groups. Transfected COS-7 cells labeled by indirect immunofluorescence (using secondary antibodies bearing **12**) were also imaged. The same filament thickness analysis yielded significantly larger values of 22 and ∼30 nm for the nanobody and antibody labeling, respectively. This comparison highlights the impact of the labeling method on the linkage error. To ensure that the difference in observed thickness was independent of the fluorophore used (**11** vs **12**), we additionally performed MINFLUX measurements of vimentin filaments prepared via GCE and stained with compound **11**, and observed a small improvement in localization precision for **12** (Figure S16b) but no significant difference in filament thickness (Figure S19a). We also observed no difference in filament thickness between transfected and wild-type COS-7 cells labeled by indirect immunofluorescence (Figure S19b). Moreover, a similar localization precision was observed when employing immunofluorescence strategies (Figure S16c,d), highlighting that the differences in observed filament thickness arose solely from linkage error.

We however note that when screening anti-vimentin antibodies on transfected cells, we failed to uniformly mark the same filaments labeled with GCE (Figure S20), serendipitously revealing two populations of filaments. The GCE strategy that we used for UAA incorporation in vimentin additionally appends a C-terminal FP, in order to mark transfected filaments and facilitate imaging. We suspect the FP, rather than the UAA, affected specificity of the primary antibodies as such modifications to vimentin are known to affect filament morphology.^57^ While beyond the scope of this work, the immunofluorescence results highlight the challenges of GCE, the design of suitable plasmids for UAA incorporation, and the discrepancies that may result from heterogeneous protein populations.

The direct incorporation of the fluorophores into the head domain of the vimentin monomers can additionally reveal information about the multimeric structure of the mature filaments. By measuring the peak-to-peak distance of localization clusters along the filament length (Figure 6d), we observed an abundance of pairwise distances of 11 nm, consistent with previous observations on the structure of vimentin unit length filaments (ULFs) utilizing C and N-terminal tagging strategies,^56^ where values of ∼39 nm and ∼10 nm are expected for separations of head domains within single ULFs and between annealed ULFs in matured filaments, respectively (Figure 6e). However, in previous work, these values could not be directly observed *in cellulo* due to limitations to resolution and linkage error, further highlighting the power of our strategy. In our case, direct tagging of N116 shifts the fluorophore position inward, increasing the observed value, but low fluorophore density and ULF orientations further contribute to measurement errors. The additional peak at 33 nm may thus be due to the ULF head domain separations. Further improvements to UAA incorporation^25,58^ as well as 3D imaging modalities can afford an even more complete visualization the of vimentin substructure.

## Conclusion

We show, for the first time, how the excited state quenching inherent to tetrazine dyads can be additionally utilized to impart chemical control over the activation process of fluorophores for next-generation super-resolution fluorescence microscopy. We have prepared a family of highly compact, biorthogonal, live-cell compatible, and photoactivatable probes for copper-free click chemistry with UAAs introduced by genetic code expansion that can be applied in STED, PALM, and MINFLUX nanoscopies. Combined with the ultimate resolution of MINFLUX, we visualized the substructure of vimentin filaments incorporating an UAA in the head domain of the filament monomers *in cellulo* and with molecular-scale precision. Our work demonstrates the combined potential of linkage-error-free labeling with appropriately designed caging-group-free photoactivatable fluorophores.

## Supporting Information Available

Additional experimental details, materials, methods, and characterization data for all new compounds (PDF)
